# A Role for the Non-Canonical Wnt-β-Catenin and TGF-β Signaling Pathways in the Induction of Tolerance during the Establishment of a *Salmonella enterica* Serovar Enteritidis Persistent Cecal Infection in Chickens

**DOI:** 10.3389/fvets.2015.00033

**Published:** 2015-09-08

**Authors:** Michael H. Kogut, Ryan J. Arsenault

**Affiliations:** ^1^Southern Plains Agricultural Research Center (SPARC), Agricultural Research Service (ARS), United States Department of Agriculture (USDA), College Station, TX, USA

**Keywords:** *Salmonella*, chickens, kinome array, Wnt signaling pathway, tolerance

## Abstract

Non-typhoidal *Salmonella enterica* induce an early pro-inflammatory response in chickens. However, the response is short-lived, asymptomatic of disease, resulting in a persistent colonization of the ceca, and fecal shedding of bacteria. The underlying mechanisms that control this persistent infection of chickens by *Salmonella* are unknown. Recently, we found an expansion of the Treg population and subsequent increased *in vitro* immunosuppressive functions of the CD4^+^CD25^+^ cells isolated from the ceca of the *Salmonella*-infected chickens by day 4 post-infection that increased steadily throughout the course of the 14 days of infection, whereas the number of CD4^+^CD25^+^ cells in the non-infected controls remained steady throughout the study. CD4^+^CD25^+^ cells from cecal tonsils of *S. enteritidis*-infected birds had greater expression of IL-10 mRNA content than the CD4^+^CD25^+^ cells from the non-infected controls at all the time points studied. These results suggest the development of a tolerogenic immune response in the cecum of *Salmonella*-infected chickens may contribute to the persistance of *Salmonella* cecal colonization. Using a chicken-specific kinome peptide immune array, we have analyzed the signaling pathways altered during the establishment of this tolerogenic state. This analysis has revealed a role for the non-canonical Wnt signaling pathway in the cecum at 4 days post-infection. Infection induced the significant (*p* < 0.01) phosphorylation of the G-protein-coupled transmembrane protein, Frizzled 1 (FZD1), resulting in an influx of intracellular Ca^2+^ and the phosphorylation of the Ca^2+^-dependent effector molecules calcium/calmodulin-dependent kinase II (CamKII), β-catenin, protein kinase C, and the activation of the transcription factor, NFAT. Nuclear translocation of NFAT resulted in a significant increase in the expression of the anti-inflammatory cytokines IL-10 and TGF-β. Increased expression of TGF-β4 mRNA activates the TGF-β signaling pathway that phosphorylates the receptor-activated Smads, Smad2 and Smad3. Combined with the results from our Treg studies, these studies describe kinome-based phenotypic changes in the cecum of chickens during *Salmonella* Enteritidis infection starting 4 days post-infection that leads to an anti-inflammatory, tolerogenic local environment, and results in the establishment of persistent intestinal colonization.

## Introduction

Salmonellosis is a zoonotic disease produced by the Gram-negative enteric bacterium *Salmonella*. *Salmonella* are not restricted to particular host species, with more than 2500 serotypes having been described mostly belonging to the species *Salmonella enterica* (1), with most having asymptomatic colonization of the gastrointestinal tract of animals. The most prevalent serovars, *S. enterica* serovar Typhimurium (*S*. Typhimurium) and serovar Enteritidis (*S*. Enteritidis) are major causes of intestinal infections in a wide range of host species worldwide (2, 3). Both serovars have a broad host range able to infect poultry, livestock, and humans (4). *S*. Typhimurium and *S*. Enteritidis infections of humans, cattle, and pigs cause self-limiting gastroenteritis manifested by abdominal pain, vomiting, and inflammatory diarrhea (5); whereas, infection of birds more than a few days old with either serovar produces asymptomatic cecal colonization with persistent shedding of bacteria that may persist for months, causing carcass contamination at slaughter with potential human food safety issues (6–10).

The host responds to infection with pathogens by activating the innate and adaptive immune mechanisms. However, some pathogens, such as *Salmonella*, have evolved the ability to survive the initial host immune response and persist. The interactions between the host and pathogen during this persistent phase are multifaceted and reflect the co-evolution of bacterial virulence mechanisms and host immune responses. Very little is known about the regulatory interactions between the host immune response and virulence mechanisms that lead to *S. enterica* persistence in the avian intestine. Chronic colonization of the intestinal tract is an important aspect of persistent *Salmonella* infection in poultry because it results in propagation of bacteria in the birds due to the impossibility to isolate contaminated animals (11).

A better comprehension of the host factors that are exploited by the bacteria in order to establish a persistent infection would be invaluable for the identification and development of therapeutic targets. Recently, Chausse and colleagues (12) found that genes involved in the inflammatory response were down-regulated during the carrier state, suggesting a bias toward a Th2 response in susceptible chickens. Furthermore, in a murine model of long-term S*. typhimurium* infection, the bacteria preferentially associated with anti-inflammatory/M2 macrophages during the later stages of infection (13). Lastly, the immune-suppression role of regulatory T cells has been shown to play a role in *Salmonella* persistence in a murine model (14). All told, we speculate that the bacterium is involved in redirecting the host response toward immune tolerance. The present study was designed to address the question on the induction of immune tolerance during a persistent paratyphoid *Salmonella* infection in chickens.

When considering the effects of an infection on a host, such as asymptomatic salmonellosis in poultry, studying the protein level as opposed to the gene or transcript level reduces complicating variables. The proteome contains the final effectors resulting in the organism’s phenotype. Such studies can provide a dramatically different perspective on the avian host’s biochemical and physiological properties to this asymptomatic enteric bacterial pathogen. Our recent report of the development of chicken species-specific peptide arrays for kinome analysis of host signaling responses to *Salmonella* provided us with the prospect to characterize a more detailed understanding of the host–pathogen interactions in the chicken (15). Using a metabolism kinome array, we have documented altered metabolic signaling pathways in the skeletal muscle of *S*. Typhimurium-infected chickens that affected fatty acid and glucose metabolism through the AMPK and the mTOR signaling pathways over the first 3 weeks post-infection (16). Additionally, using a chicken-specific immune array, we have detailed the toll-like receptor (TLR) signaling pathways stimulated in monocytes by TLR ligands, CpG (TLR 21) and poly I:C (TLR3), but also identified a unique signaling pathway stimulated by the combination of CpG/poly I:C treatment that was not observed by treatment with the individual ligands (17).

Therefore, in the present study we hypothesized that *S. enterica* serovar Enteritidis (*S*. Enteritidis) induces an immune tolerance in chickens that results in the bacteria’s ability to persistently colonize the cecum of poultry. To test this hypothesis, we analyzed temporal chicken-specific kinomic immune peptide arrays anti-inflammatory cytokine gene transcription of avian cecal tissue during a persistent infection by *S*. Enteritidis. Using these approaches, we were able to begin to characterize the specific immune post-translational signaling events during a persistent *Salmonella* colonization in chickens. Furthermore, we characterized the cellular and cytokine profiles that provide confirmation for the transition of an early pro-inflammatory mucosal response to the development of an immune tolerogenic mucosal response.

## Materials and Methods

### Experimental animals

Experiments were conducted according to the regulations established by the U.S. Department of Agriculture Animal Care and Use Committee. Straight-run broiler chickens used in this study were obtained from a commercial breeder and were all of the same genetic background and were not vaccinated at any time. Chicks were placed in floor pens containing wood shavings, provided supplemental heat, water, and a balanced, unmedicated corn and soybean meal-based chick starter diet *ad libitum* that met or exceeded the levels of critical nutrients recommended by the National Research Council (18). *Salmonella* was not detected in the feed or from the paper tray liners using standard procedures (19).

### *S*. enteritidis challenge

A poultry isolate of *S. enterica* serovar Enteritidis [*S*. Enteritidis; (ID 9711771, part 24)] was obtained from the National Veterinary Services Laboratory (Ames, IA, USA), and was selected for resistance to nalidixic acid and novobiocin and maintained in tryptic soy broth (Difco Laboratories, Sparks, MD, USA) containing antibiotics (20 μg/ml nalidixic acid and 25 μg/ml novobiocin; Sigma Chemical Co., St. Louis, MO, USA). A stock culture was prepared in sterile PBS and adjusted to a concentration of 1 × 10^9^ colony forming units (cfu)/ml. The viable cell concentration of the challenge dose for each experiment was determined by colony counts on XLT4 agar base plates with XLT4 supplement (Difco) and nalidixic acid and novobiocin (XLT-NN).

### Experimental design

One-day-old broiler chickens were randomly distributed into either non-infected control or infected groups each with 50 birds per group. The birds were fed a balanced, unmedicated corn and soybean meal-based diet. At 4 days post-hatch, all chickens were orally challenged with 1 ml of either 5 × 10^6^ CFU/ml *S*. Enteritidis or mock challenged with 1 ml sterile PBS. Four, 7, 10, and 14 days after challenge, 10 chickens from each group were humanely euthanized, cecal contents were analyzed for *S*. Enteritidis colonization, cecal tonsils were collected for quantitative real-time PCR (qRTPCR), and cecal tissue from 3 of the 10 chickens per treatment was flash frozen in liquid nitrogen and stored for use in the peptide arrays (see below).

All experiments were replicated three times. Therefore, for the mRNA expression, the ceca from a total of 40 chickens for each of the two groups (10 chickens at each of four time points) were used to prepare the mRNA for the qRT-PCR assays. RNA from each bird (*n* = 10) was isolated and assayed separately and not pooled. Each RNA sample was replicated three times per immune gene per experiment).

### Sample collection for peptide and antibody arrays

At 4, 7, 10, and 14 days post-infection, a 25 cm^2^ section from one cecum (from the middle of the cecal pouch) was removed from each of the three randomly selected birds from each group (non-infected and infected) and immediately flash frozen in liquid nitrogen to preserve kinase enzymatic activity, and then transferred to a −80°C freezer until used in the peptide array. Following microbiological analysis of the cecal contents (see below), the cecal tissues from three confirmed non-infected and three confirmed infected chickens (out of the 10 birds per group peer time point) were used for the peptide and antibody arrays.

### Peptide arrays

At each of the time points and under each condition (infected and uninfected), three cecal samples from three different animals were taken from storage for analysis (24 samples total). Cecal tissue samples were weighed to obtain a consistent 40 mg sample for the array protocol. Samples were homogenized in lysis buffer and the homogenates were used in the peptide array protocol as described previously (19, 20).

### Antibody array

The Wnt pathway antibody array assay kit was obtained from Full Moon BioSystems (Sunnyvale, CA, USA) and the protocol was carried out as per the manufacturer’s instructions. The antibody array was used as an alternative to performing several western blot assays.

### Data analysis for peptide and antibody arrays

Data normalization and PCA analysis were performed for both the peptide and antibody microarrays as described previously (21). Gene ontology (GO) and Kyoto encyclopedia of genes and genomes (KEGG) pathway analysis were performed by uploading the statistically significant peptide lists to the search tool for the retrieval of interacting genes (STRING) (22).

### Sample collection for bacterial contents

The ceca from each chicken were removed aseptically and the contents (0.25 g) from one cecal pouch was serially diluted in sterile saline to 1:100, 1:1000, or 1:10,000 and spread onto XLT-NN plates. The plates were incubated at 37°C for 24 h, and the number of NN-resistant *S*. Enteritidis cells per gram of cecal contents was determined. The data from each experimental group were pooled from three separate trials for statistical analysis.

### Sample collection for mRNA

Chickens from each experimental group were euthanized at 4, 7, 10, and 14 days post-infection. A 25-mg piece of tissue was removed from the cecal tonsils and was washed in PBS, placed in a 2-ml microcentrifuge tube with 1 ml of RNAlater (Qiagen, Inc., Valencia, CA, USA), and stored at −20°C until processed. In addition, for comparison purposes, a 25 mg piece of ceca from the 10 extra birds from each group (see above 50 birds total per group, 10 used per time point = 40) was collected at 2 days post-infection.

### RNA isolation

Tissues (25 mg) were removed from RNAlater and transferred to pre-filled 2 ml tubes containing Triple-Pure™ 1.5 mm zirconium beads. RLT lysis buffer (600 μl) from the RNeasy mini kit (Qiagen) was added and the tissue was homogenized for 1–2 min at 4,000 rpm in a Bead Bug microtube homogenizer (Benchmark Scientific, Inc., Edison, NJ, USA). Total RNA was extracted from the homogenized lysates according to the manufacturer’s instructions, eluted with 50 μl RNase-free water, and stored at −80°C until qRT-PCR analyses were performed. RNA was quantified and the quality evaluated using a spectrophotometer (NanoDrop Products, Wilmington, DE, USA). Total RNA (300 ng) from each sample was prepared.

### Quantitative real-time PCR

Primer and probe sets for the cytokines and 28S rRNA were designed using the Primer Express Software program (Applied Biosystems, Foster City, CA, USA) have been described (23, 24) and are provided in Table 1. The qRT-PCR was performed using the TaqMan fast universal PCR master mix and one-step RT-PCR master mix reagents (Applied Biosystems). Amplification and detection of specific products were performed using the Applied Biosystems 7500 Fast real-time PCR system with the following cycle profile as described previously (23, 24). Quantification was based on the increased fluorescence detected by the 7500 Fast sequence detection system due to hydrolysis of the target-specific probes by the 5-nuclease activity of the r*Tth* DNA polymerase during PCR amplification. Normalization was carried out using 28S rRNA as a housekeeping gene. To correct for differences in RNA levels between samples within the experiment, the correction factor for each sample was calculated by dividing the mean threshold cycle (*CT*) value for 28S rRNA-specific product for each sample by the overall mean *CT* value for the 28S rRNA-specific product from all samples. The corrected cytokine mean was calculated as follows: (average of each replicate × cytokine slope)/(28S slope × 28S correction factor). Fold changes in mRNA levels were calculated from mean 40 *CT* values by the formula 2^(40 *CT* of infected group − 40 *CT* in non-infected group)^.

**Table 1:** **Real-time quantitative RT-PCR probes and primers for IL-6 and TGF-**β**4**.

RNA target		Probe/primer sequence	Accession number^a^
28S	Probe	5′-(FAM^d^)-AGGACCGCTACGGACCTCCACCA-(TAMRA)-3′	X59733
	F^b^	5′-GGCGAAGCCAGAGGAAACT-3′	
	R^c^	5′-GACGACCGATTGCACGTC-3′	
IL-6	Probe	5′-(FAM)-AGGAGAAATGCCTGACGAAGCTCTCCA-(TAMRA)-3′	AJ250838
	F	5′-GCTCGCCGGCTTCGA-3′	
	R	5′-GGTAGGTCTGAAAGGCGAACAG-3′	
TGF-β4	Probe	5′-(FAM)-ACCCAAAGGTTATATGGCCAACTTCTGCAT-(TAMRA)-3′	M31160
	F	5′-AGGATCTGCAGTGGAAGTGGAT-3′	
	R	5′-CCCCGGGGTTGTGTGTTGGT-3′	

*^a^Genomic DNA sequence*.

*^b^Forward*.

*^c^Reverse*.

*^d^5-carboxyfluorescein*.

### Calcium detection

Intracellular Ca^2+^ in cecal lysates from non-infected and infected chickens was measured with a colorimetric Ca^2+^ Detection Kit (Abcam, Inc., Cambridge, MA, USA). Preparation of cell extracts was done according to the manufacturer’s instructions. Total amount of Ca^2+^ was determined using a standard curve.

### Statistical analysis

The data from these replicated experiments were pooled for presentation and statistical analysis. The mean and SEM were calculated and differences between groups were determined by analysis of variance. Significant differences were further separated using Duncan’s multiple-range test (23). A *p* value of <0.05 was considered statistically significant.

## Results

### *S*. enteritidis infection

Infection status of the chickens was confirmed by *S*. Enteritidis culturing of cecal contents and feces from each bird with and without enrichment. Cultures confirmed that greater than 70% of the chickens in the infected group displayed *S*. Enteritidis throughout the experiment while *Salmonella* was not isolated from the birds in the control group (Tables 2 and 3).

**Table 2:** **Number of chickens positive for *Salmonella* Enteritidis ceca colonization for 2 weeks following challenge**.

	Percent positive for *Salmonella* Enteritidis cecal colonization (total positive/total challenged)			
	Days post-challenge			
Treatment groups	4	7	10	14
Non-infected control	0 (0/30)	0 (0/30)	0 (0/30)	0 (0/30)
Infected	100 (30/30)	100 (30/30)	85 (26/30)	70 (21/30)

*Results shown are pooled from three separate experiments*.

**Table 3:** **Cecal *Salmonella* Enteritidis for 2 weeks following challenge**.

	CFU of *Salmonella* Enteritidis in cecum (log 10)			
	Days post-challenge			
Treatment groups	4	7	10	14
Non-infected control	0	0	0	0
Infected	4.11 ± 1.37	4.79 ± 1.28	5.23 ± 1.41	4.36 ± 1.78

*Results are expressed as the mean ± SEM of three separate experiments*.

### Altered expression of cytokines

**Figure 1:**
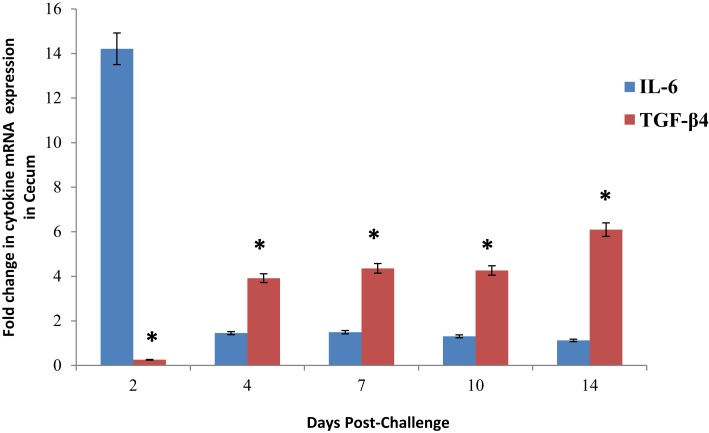
**Expression of pro-inflammatory (IL-6) or anti-inflammatory TGF-**β**4) cytokine mRNA in the ceca from experimental chickens with persistent colonization by *Salmonella* Enteritidis**. The expression of cytokine mRNA was determined by quantitative RT-PCR. Data represent the fold change in mRNA expression in the cecum from infected chickens when compared to the mRNA expression in the cecum from non-infected chickens. Data represent the mean ± SEM from three separate experiments.

Characteristically, during an acute infection (within 24 h) by paratyphoid strains of *Salmonella* in chickens, there is an up-regulation of pro-inflammatory cytokines mRNA expression in the cecum (25). Few, if any, studies have measured the comparative expression of mRNA between anti-inflammatory cytokines and pro-inflammatory cytokines during persistent *Salmonella* infections. In the present studies, we profiled the pro-inflammatory (IL-6) and anti-inflammatory (TGF-β4) cytokine mRNA expression in the cecum of chickens over the first 14 days post-infection with *S*. Enteritidis and compared the results to the non-infected control birds. Initially, at 48 h post-infection, as expected, IL-6 (14.21-fold change) mRNA expression in the ceca from *S*. Enteritidis-infected chickens was up-regulated when compared to the expression in the cecum from the non-infected birds (Figure 1). However, by 4 days post-infection, there was a dramatic reduction in IL-6 mRNA expression (1.45-fold change) that remained low throughout the 14 days post-infection. In fact, there was no statistical difference in IL-6 mRNA expression between the infected and non-infected cecal tissues.

Alternatively, as the pro-inflammatory IL-6 mRNA expression was decreasing, the *S*. Enteritidis infection persisted, and the expression of the anti-inflammatory cytokine TGF-β4 mRNA were significantly up-regulated over the course of the 14-day infection period with the most profound up-regulation of both between 7 and 10 days post-infection when compared to the non-infected controls birds (Figure 1).

### Peptide arrays

Chicken-specific peptide arrays designed for the study of chicken immune signaling pathways were used to analyze the cecal samples from the non-infected and infected control chickens. To account for any changes in phosphorylation state that were not due to the infection, the results at each time point were corrected using their respective time matched controls.

The KEGG pathway results generated from STRING showed a large number of pathways implicated by the data at a statistically significant level [*p* < 0.05 false discovery rate (FDR) corrected]. Of particular interest were those pathways that showed a large number of statistically significant peptides that were phosphorylated at different times over the course of the study. These pathways are shown in Table 4. Of note are the Wnt signaling and TGF-β4 signaling pathways that were dramatically altered by the infection. Both of the pathways had multiple significantly altered peptide phosphorylation events at multiple time points post-infection; however, a total of 20 differentially phosphorylated peptides were found within these two pathways in chickens on the fourth day post-infection with *S*. Enteritidis (Table 4), signifying a dramatic local post-translational modification of the infected cecum. Of the 20 peptides that were differentially phosphorylated, 13 belong to the Wnt signaling pathway and 7 to the TGF-β4 pathway. Interestingly, only nine more total peptides were found to be differentially phosphorylated within these two specific pathways over days 7–14 post-infection (Table 4).

**Table 4:** **KEGG pathways generated by STRING**.

		4 days		7 days		10 days		14 days	
GO ID	Pathway	# peptides	*p*-Value (FDR)	# peptides	*p*-Value (FDR)	# peptides	*p*-Value (FDR)	# peptides	*p*-Value (FDR)
hsa04141	Protein processing in endoplasm reticulum pathway	4	0.07	13	0.073	1	1.00	3	0.071
hsa05130	Pathogenic *Escherichia coli* infection	–	N/S	2	N/S	4	N/S	4	N/S
hsa04250	TGF-β4 signaling pathway	12	0.016	–	N/S	–	N/S	–	N/S
hsa04310	Wnt signaling pathway	16	0.0004	2	N/S	–	N/S	5	0.024
hsa04623	Cytosolic DNA-sensing pathway	4	7.01 × 10^−2^	5	N/S	4	N/S	–	N/S
hsa05217	Basal cell carcinoma	–	N/S	–	N/S	2	0.338	–	N/S
hsa04672	Intestinal immune response for IgA production	1	1.00	5	N/S	–	N/S	–	N/S

*Peptides that displayed a significant change in phosphorylation state were input into the STRING database for each time point. Generated pathways involved in immune activation/suppression that displayed *p*-value of less than 0.05 (FDR corrected) are listed. N/S indicates that the pathway is non-significant*.

### Phosphorylation events within specific pathways

#### Wnt Signaling Pathway

Frizzled is a family of G protein-coupled receptor proteins that serves as receptors in the Wnt signaling pathway. We found frizzled 1 (FZD1) to be significantly phosphorylated in the ceca of *S*. Enteritidis-infected chickens (Table 5), providing us with the first indication of the involvement of the Wnt pathway. Further analysis of the peptide alterations in the Wnt signaling pathway revealed other significant changes in phosphorylation events: (1) the serine/threonine kinase, glycogen synthase kinase 3β (GSK3β), was significantly dephosphorylated in the *S*. Enteritidis-infected cecal tissue at 4 days post-infection when compared to the non-infected control cecal tissue, while β-catenin was significantly phosphorylated; (2) both protein kinase C (PKC) alpha isoforma (PRKCA) and calcium/calmodulin-dependent protein kinase type II alpha chain (CaMK2A) are significantly phosphorylated in the *S*. Enteritidis-infected cecal tissue at 4 days post-infection when compared to the non-infected control cecal tissue; (3) the serine–threonine protein calcineurin (PPSCA), an inhibitor of the canonical Wnt signaling pathway, was significantly phosphorylated in the *S*. Enteritidis-infected cecal tissue at 4 days post-infection; and (4) nuclear factor of activated T-cells (NFAT), a family of transcription factors that play a pivotal role in the transcription of cytokine genes and other genes critical for the immune response were found to be significantly phosphorylated in the ceca of *S*. Enteritidis-infected chickens (Table 5).

**Table 5:** **Peptides from the Wnt signaling pathway that displayed a statistically significant change in phosphorylation**.

	Wnt signaling pathway							
	Days post-infection							
	4		7		10		14	
Peptide	Fold change	*p*-Value	Fold change	*p*-Value	Fold change	*p*-Value	Fold change	*p*-Value
CAMK2A	2.789	0.008	–		–		–	
β-catenin	1.657	0.044	1.538	0.035	–		–	
EP300	1.954	0.017	–		–		1.804	0.047
Jun	3.604	0.001	–		–		–	
Jun	3.389	0.0003	–		–		–	
GSK-3β	−2.254	0.002	–		2.448	0.018	–	
NFATC1			–		–		1.957	0.009
NFATC2	1.779	0.045	–		–		–	
NFATC3	2.239	0.006	–		–		1.655	0.020
Calcineurin	2.447	0.003	–		–		–	
PRKCA Thr638	1.744	0.042	–		–		–	
PRKCA Tyr657	2.555	0.011	–		–		1.927	0.024
RAC1	1.740	0.008	–		–		–	
SMAD2 Ser345	3.434	0.008	1.756	0.013	–		–	
SMAD2 Thr255	4.532	0.006	–		–		–	
SMAD3	1.442	0.033	–		–		–	
FZD1	2.712	0.034	–		–	–	1.844	0.008

*Peptides that displayed a *p*-value of less than 0.05 are listed*.

#### TGF-β Signaling Pathway

The TGF-β signaling pathway showed statistically significant changes in the cecal tissue from chickens 4 days post-infection with *S*. Enteritidis (Table 6). Smad proteins 1–3 were all significantly phosphorylated in the infected cecal tissue. Smads are intracellular proteins that transduce extracellular signals from TGF-β ligands and activate gene transcription. Smads 1–3 are receptor-regulated proteins that associate with receptor kinases and are phosphorylated. These proteins then typically bind to the common mediator Smad or co-Smad, Smad4. Smad complexes then accumulate in the cell nucleus where they regulate transcription of specific target genes.

**Table 6:** **Peptides from the TGF-**β**4 signaling pathway that displayed a statistically significant change in phosphorylation**.

	TGF-**β**4 signaling pathway							
	Days post-infection							
	4		7		10		14	
Peptide	Fold change	*p*-Value	Fold change	*p*-Value	Fold change	*p*-Value	Fold change	*p*-Value
EP300	1.954	0.017	–		–		–	
MAP3K7 (TAK1)	2.709	0.016	–		–		–	
MAPK8 (JNK1)	2.207	0.022	–		–		1.167	0.013
MAPK3 (ERK1)	4.269	0.001	–		–		–	
p70S6K	1.145	0.023	–		–		–	
P70S6K	3.005	0.012	–		–		–	
P70S6K	3.759	0.009	–		–		–	
SMAD1	2.698	0.021	–		–		–	
SMAD1	2.916	0.00	–		–		–	
SMAD2 Ser345	3.434	0.008	–		–		–	
SMAD2 Thr255	4.532	0.006	–		–		–	
SMAD3	1.441	0.032	–		–		–	

*Peptides that displayed a *p*-value of less than 0.05 are listed*.

In addition to Smad signaling, which directly impacts transcription, TGF-β induces mTORC1 (the mammalian target of rapamycin complex 1) signaling through phosphoinositide 3-kinase (PI3K) and Akt (26). Further analysis of the TGF-β signaling pathway showed a significant phosphorylation of p70S6 kinase, a serine–theonine kinase that is a target for the S6 ribosomal protein (Table 6). P70S6 kinase is in a signaling pathway that includes the mammalian target of rapamycin (mTOR). mTOR can be activated in distinct ways, thereby activating p70S6K.

### Validation of kinome analysis with antibody array

Despite the scarcity of chicken-specific antibodies, the key proteins of interest based on the peptide array results were relatively well conserved between humans and chickens, giving us confidence that we would observe significant cross-reactivity from the antibodies. The percent orthology between the human and chicken at the 15 amino acid phosphorylation target sites as determined by NCBI Protein Blast analysis is shown in Table 7. Following the data normalization, the results pointed to a similar pattern to that observed with the peptide arrays (Table 7).

**Table 7:** **antibody array results**.

	Aantibody array		Peptide array			Homology
ID	Fold change	*p*-value	ID	Fold change	*p*-value	
Calmodulin (phospho-Thr286)	1.31	0.003	Calmodulin T185	2.99	0.008	100
CAMK2-beta (phospho-Ser33)	1.18	0.002	CAMK2-beta Ser33	1.66	0.044	100
MAP3K7 (TAK1) (phospho-431)	1.49	0.001	MAP3K7 (TAK1) Ser446	2.71	0.016	100
MAP3K7 (TAK1) (phospho-Thr187)	1.50	0.005	MAP3K7 (TAK1) T177	1.85	0.05	100
NFATC2 (phospho-Ser168/170)	1.05	0.009	NFATC2	1.76	0.045	100
NFATC4 (phospho-Ser203)	2.21	0.006	NFATC4 Ser203	2.24	0.01	100
PPP2CA (phospho-Ser307)	1.91	0.03	PPP2CA Ser304	2.45	0.003	93
SMAD1 (phospho-Ser206)	2.70	0.021	SMAD1 Ser206	2.09	0.02	100
SMAD1 (phospho-Ser462)	2.92	0.004	SMAD1 Ser462	3.43	0.008	100
SAMD2 (phospho-Thr220)	1.68	0.025	SMAD2 Thr255	4.53	0.006	93
SMAD2 (Ser350)	1.21	0.004	SMAD2 Ser345	3.43	0.008	100
SAMD3 (phospho-Thr199)	1.36	0.001	SAMD3 Thr180	1.44	0.006	93
PKCA (phosphor-Thr640)	1.18	0.036	PKCA Thr640	2.55	0.01	100
PKCA (phospho-Ser657)	1.34	0.0009	PKCA Ser659	1.74	0.04	100
PLCB (phospho-Tyr783)	−1.19668	0.03417	PLCG1 Y675	−2.78738	0.00067	86

*Statistically significant (*p* < 0.05) phosphospecific antibody array results of Salmonella Entertidis cecal samples. Four days post-infection samples were compared to non-infected control samples to find changes in infected cecal tissue over time. Antibodies bound to phosphorylated protein having a statistically significant difference in fluorescent signal are shown. Fold Change Antibody Array is the change in fluorescent signal when comparing the infected samples to control samples. Homology indicates the % similarity between human and chicken at the 15 amino acid region flanking the phosphorylation residue. Fold Change Peptide Array is the change in fluorescent signal as indicated by the peptide array*.

### Verification of increased Ca^2+^ in infected cecal tissues

**Figure 2:**
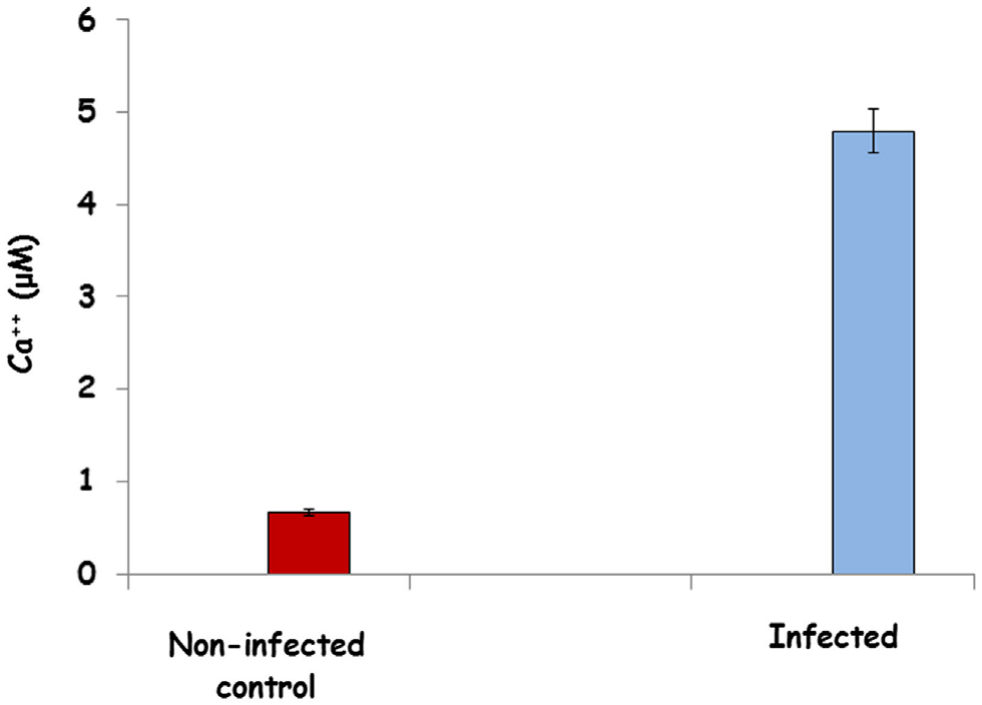
**Ca^2+^ levels in the ceca of *Salmonella*-infected and non-infected chickens 4 days post-challenge**. Intracellular Ca^2+^ in cecal lysates from non-infected and infected chickens was measured with a colorimetric Ca^2+^ Detection Kit. Data represents the amount of Ca^2+^ measured in the cecal tissue from infected and non-infected chickens and are expressed as the mean micromoles of Ca^2+^ ± SEM from three separate experiments.

Using a commercial ELISA kit to measure intracellular Ca^2+^ levels, we found almost five times more intracellular Ca^2+^ in the cecal tissue from chickens 4 days post-infection with *S*. Enteritidis (Figure 2). These results provide further proof of the activation of the non-canonical Wnt-Ca^2+^ signaling pathway during the establishment of a persistent *Salmonella* infection in the cecum.

## Discussion

In chickens, *Salmonella* have evolved the capacity to survive the initial immune response and persist. Very little is known about the regulatory interactions between the host immune response and virulence mechanisms that lead to *S. enterica* persistence in the avian intestine. The carrier state, corresponding to a persistent colonization of the gut, is established, and *Salmonella* is able to stay in the ceca for months without clinical signs (10). Chronic colonization of the intestinal tract is an important aspect of persistent *Salmonella* infection because it results in a silent propagation of bacteria in poultry stocks due to the impossibility to isolate contaminated animals (11).

Collectively, the results from the current experiments demonstrate the phenotypic plasticity of the avian immune system in the gastrointestinal tract as it first orchestrates an inflammatory response against a primary *Salmonella* infection followed by a dramatic change in the immune microenvironment during the establishment of a persistent *Salmonella* infection.

The 4-day post-infection time period is the initiation of a transitional period between the acute inflammatory response to a primary *Salmonella* infection and the establishment of an “immune *status quo*” (27). As described in the present experiments, by 4 days post-infection, we see a dramatic down-regulation of pro-inflammatory cytokine expression that coincides with the up-regulation of anti-inflammatory cytokine expression (Figure 1). Further, by day 4 post-infection, a dramatic increase in Tregs (CD4^+^CD25^+^) in the cecum and remains elevated through the 14-day post-infection time period (27). This coordinated production of pro- versus anti-inflammatory responses is fundamental for the development of an effective initial inflammatory response and subsequent return to tissue homeostasis. Finally, we used the power of kinomics to highlight the mechanisms used by *S*. Enteritidis to alter the avian inflammatory responses and uncover host signaling events that are manipulated by the bacteria in order to establish a persistent infection. Our experiments have identified multiple effects on the host kinome during the establishment of a *Salmonella* persistent infection in the avian cecum. This comparative immune kinome analysis between the *S*. Enteritidis-infected avian cecum versus non-infected cecum provides unique information on host molecular signaling cascades that are mobilized during the establishment of *Salmonella* persistence. Additionally, the relative lack of differential phosphorylation events found in the host signaling pathways between the infected and non-infected ceca 7–14 days post-infection are suggestive that a level of homeostasis was achieved and that the *Salmonella* were no longer recognized as “foreign” and were part of the commensal population. Future experiments are planned to characterize and compare this homeostasis to that of the non-infected controls. Lastly, the identified tissue protein kinases represent potential targets for future antimicrobial compounds for decreasing *Salmonella* loads from the intestines of food animals before going to market.

### TGF-β signaling pathway

The purpose of these studies was to begin to understand and characterize the biological and molecular mechanisms that regulate the mucosal phenotype of the chicken cecum during the establishment of a persistent infection by *Salmonella*. CD4^+^CD25^+^ regulatory T cells (Tregs) are potent regulators of immune homeostasis (28). We have recently described a dramatic increase in the number of Tregs in the chicken cecum 4 days post-infection with *Salmonella* Enteritidis, and that the number of T regs remains elevated through the 14 days post-infection (29). Understanding the signals necessary for this generation and expansion of Tregs is important for understanding persistence of *Salmonella* in poultry. The pleotropic cytokine, TGF-β, plays a major role in the regulation of inflammation, with T cells being a key target (30). TGF-β suppresses T cell proliferation and T effector cell function (30) while promoting the generation and function of Treg cells (31). Here, we show a dramatic increase in TGF-β mRNA expression between 2 and 4 days after *Salmonella* challenge that remained elevated through 14 days post-infection (Figure 1). Simultaneously, using our chicken-specific immune kinome array, for the first time, we have characterized dramatic changes in phosphorylation events where we observed a significant increase in phosphorylation events within the TGF-β signaling pathway (Table 6). The TGF-β signaling pathway is mediated by a Smad transcription factor-dependent pathway (21). Upon ligand binding, the TGF-β receptor phosphorylates and activates receptor-associated Smad2 and Smad3, which then associate with Smad4 to control TGF-β-targeted gene expression (32). Both Smad2 and Smad3 are significantly phosphorylated in the ceca from *Salmonella*-infected chickens at 4 days post-infection (Table 6). Mitogen-activated protein kinases can also mediate TGF-β signaling through Smad-independent pathways, including TGF-β-activated kinase (TAK1 and MAP3K7), extracellular signal-regulated kinase (ERK and MAPK3), Jun-N-terminal-3-kinase (JNK and MAPK8) (33, 34), all of which are significantly phosphorylated in the ceca from the *Salmonella*-infected chickens at 4 days post-infection. Therefore, the establishment of a persistent cecal colonization in chickens by *Salmonella* initiates the activation of both the canonical (Smad-dependent) and non-canonical (Smad-independent) TGF-β signaling pathways. Smad-dependent and -independent TGF-β signaling appear to separately control Treg and non-Treg function (35). Specifically, Smad-dependent pathways appear to be required to mediate TGF-β functions in non-Treg cells, including non-T cells, whereas Smad-independent pathways are important for Treg function (35, 36). Further, Smad 2/3 are involved in the suppression of pro-inflammatory cytokines by inhibiting the activation of different signal transducers and activators of transcription (STAT) proteins, including STAT1 and 4 to inhibit IFN-γ production (37). Therefore, it is reasonable to speculate that the change in the cecal mucosal phenotype from pro-inflammatory to tolerance is at least partially mediated by the increased expression of TGF-β that results in the activation of both Smad-dependent and -independent TGF-β pathways and the increase differentiation and function of Tregs that provide the environment essential for *Salmonella* to establish a persistent infection.

### Wnt signaling pathway

The Wnt signaling pathway system is evolutionarily conserved system that regulates a diverse series of essential functions (38, 39). There are three distinct pathways in Wnt signaling: the canonical Wnt/β-catenin and two non-canonical pathways, Wnt/Planar Cell Polarity and Wnt/Ca^2+^ pathways (39). Based on the results from the kinome array, a number of peptides from the Wnt signaling pathway exhibited statistically significant changes in their phosphorylation (Table 5). Further observation of these results indicates that two of the Wnt signaling pathways had significant changes in phosphorylation events, namely the canonical Wnt/β-catenin and the non-canonical Wnt/Ca^2+^ pathways.

### Bacterial effect on canonical Wnt signaling

The phosphorylation of two peptides in the canonical Wnt pathway was significantly altered at the 4 days after *Salmonella*-infection time point: glycogen synthase kinase 3β (GSK-3β) and β-catenin (Table 5). GSK-3β was significantly dephosphorylated, whereas β-catenin was significantly phosphorylated in these experiments. GSK-3β is a constitutively active serine/threonine kinase that regulates the phosphorylation and degradation of β-catenin (40). In response to external stimuli, GSK-3β is regulated by phosphorylation, inactivated by dephosphorylation of Tyr216 or activated by dephosphorylation of Ser9. The results found here determined that GSK-3β was dephosphorylated at Ser9. This site-specific phosphorylation of GSK-3β results in the activation of its kinase activity (41). In its activated form, GSK-3β forms a catalytically active complex that phosphorylates β-catenin inducing ubiquitylation and proteasomal degradation of β-catenin (39). In these studies, β-catenin was phosphorylated at Ser33 (Tables 5 and 6). This phosphorylation site promotes ubiquitylation and targeted destruction of β-catenin (39).

### Bacterial effect on non-canonical Wnt signaling

**Figure 3:**
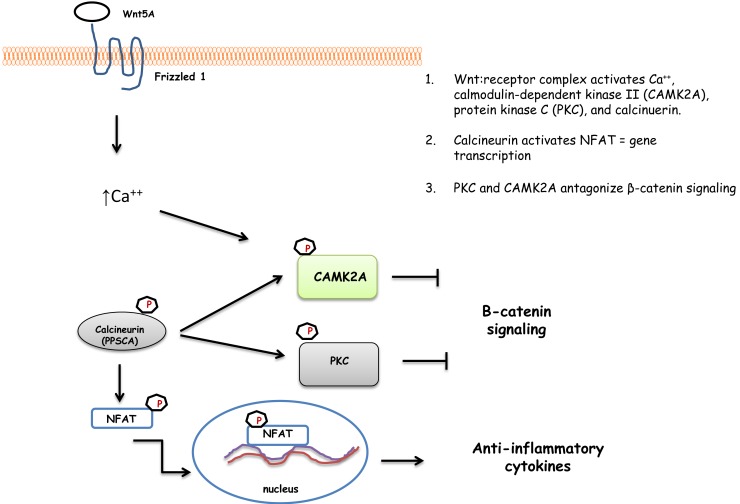
**A schematic of the proposed non-canonical Wnt signaling pathway induced in the ceca from experimental chickens with persistent colonization by *Salmonella* Enteritidis**.

Non-canonical Wnt signaling controls nuclear localization of nuclear factor of activated T cell (NFAT) transcriptional factor though Ca^2+^ and suppresses canonical Wnt signaling (42, 43). Using the chicken-specific kinome array (Table 5), Wnt antibody array (Table 6), and a Ca^2+^ ELISA assay (Figure 2), we have outlined the activation of the entire non-canonical Wnt/Ca^2+^ pathway in the cecum of chickens as *Salmonella* establishes a persistent infection beginning 4 days after infection (Figure 3). The Wnt5A (fold change = 1.82696, *p* < 0.0001)/frizzled 1 receptor complex induces the influx of intracellular Ca^2+^ that, in turn, phosphorylates calcinuerin, a Ca^2+^, calmodulin-dependent serine/threonine protein phosphatase. Phosphorylated calcinuerin phosphorylates both calmodulin-dependent kinase II (CaMK2A) and PKC and activates NFAT, which can then translocate to the nuclease to induce gene transcription (44). NFAT functions to regulate the interaction of the innate immune cells with acquired immunity and to promote anti-inflammatory programs (45). Thus, the increased phosphorylation of NFAT peptides would suggest the initiation of anti-inflammatory signals. Further, Ca^2+^/calcineurin/NFAT pathway is crucial for both development and function of regulatory T cells (46). Lastly, NF-κB (nuclear factor kappa-light-chain-enhancer of activated B cells) is a transcription factor that plays a key role in regulating the pro-inflammatory response whose activity is triggered in response to infectious agents and pro-inflammatory cytokines via the IκB kinase (IKK) complex (47, 48). Thus, dephosphorylation of both IKK (Thr37, fold change = −1.63995, *p* < 0.0024; Ser194, fold change = −2.86736, *p* < 5 × 10^5^) and NF-κB (NF-κB1 p105, Ser342, fold change = −31.2387, *p* < 0.025) would result in a down-regulation of pro-inflammatory cytokines, as we observed in the present experiments (Figure 1).

In summary, the results from the present studies, taken together with our previous studies with Treg, provide solid evidence of a phenotypic change in the mucosal microenvironment that allows for the establishment of a persistent infection by *S*. Enteritidis in the avian cecum. The phenotype alteration appears to be partially mediated due to the targeting of signaling cascades, such as the non-canonical Wnt/Ca^2+^ and the TGF-β signaling pathways that inhibit the transcription of pro-inflammatory responses that provide an appropriate local environment for the generation and expansion of Tregs.

## Conflict of Interest Statement

The authors declare that the research was conducted in the absence of any commercial or financial relationships that could be construed as a potential conflict of interest.
